# Infliximab Ameliorates Methotrexate-Induced Nephrotoxicity in Experimental Rat Model: Impact on Oxidative Stress, Mitochondrial Biogenesis, Apoptotic and Autophagic Machineries

**DOI:** 10.1007/s12013-023-01168-7

**Published:** 2023-09-01

**Authors:** Eman F. Wasfey, Marah Shaaban, Manalia Essam, Youssef Ayman, Salma Kamar, Tasneem Mohasseb, Rana Rozik, Huda Khaled, Mohamed Eladly, Mohammed Elissawi, Ahmed Bassem, Shimaa Z. Elshora, Sara M. Radwan

**Affiliations:** 1 Biochemistry Department, Faculty of Pharmacy, Ain Shams University, Cairo, Egypt; 2 Drug Design Program, Faculty of Pharmacy, Ain Shams University, Cairo, Egypt; 3 Histology Department, Faculty of Medicine for Girls, Al-Azhar University, Cairo, Egypt

**Keywords:** Infliximab, MTX-induced nephrotoxicity, PGC-1α, autophagy, Mitochondrial biogenesis.

## Abstract

Accumulating data confirms that Methotrexate (MTX), a well-known immunosuppressive and anticancer drug, causes nephrotoxicity. Infliximab (INF), the inhibitor of tumor necrosis factor-alpha (TNF-α), was proven to have anti-inflammatory properties. Thus, it may have potential in preventing MTX-induced nephrotoxicity. Therefore, this study aimed to inspect the prospective nephroprotective effect of INF on MTX-induced rat nephrotoxicity through investigating the possible molecular mechanisms, including its interference with different death routes, oxidative stress as well as mitochondrial biogenesis. Rats received an INF intraperitoneal single dose of 7 mg/kg 72 h prior to a single 20 mg/kg MTX injection. MTX nephrotoxicity was demonstrated by significantly increased serum levels of the renal indicators urea and creatinine as well as renal inflammatory markers TNF-α and Interleukin-6 (IL-6) and the renal oxidative stress marker malondialdehyde (MDA), while renal antioxidant enzyme superoxide dismutase (SOD) was significantly decreased compared to control. INF injection prior to MTX markedly reversed these MTX-induced effects. Besides, MTX impaired mitochondrial biogenesis, while INF attenuated this impairment, as indicated by increased expression of peroxisome proliferator-activated receptor-γ coactivator-1α (PGC-1α). Finally, MTX triggered apoptotic and autophagic cascades in renal tissues as evidenced by reduced anti-apoptotic Bcl-2 protein expression as well as elevated expression of the pro-apoptotic protein Bax and both key regulators of autophagy; beclin-1 and LC-3, whereas INF pretreatment counteracted these apoptotic and autophagic effects of MTX. Summarily, these results suggest that INF provides protection against MTX-induced nephrotoxicity which could be elucidated by its antioxidant, anti-inflammatory, anti-apoptotic and anti-autophagic effects as well as upregulating mitochondrial biogenesis.

## Introduction

Methotrexate (MTX) is widely used for various malignancies and several autoimmune conditions. It inhibits DNA synthesis by inhibiting the dihydrofolate reductase enzyme [1]. Unfortunately, its clinical use is restricted due to its associated nephrotoxicity [2]. MTX-induced nephrotoxicity is assumed to be concurrent with oxidative stress, inflammation, mitochondrial dysfunction and apoptosis [3, 4]. MTX induces oxidative stress; causing failure to control the levels of pro-oxidants and antioxidants. Consequently, exposure to too much Reactive Oxygen Species (ROS) causes cell structure damage [5].

Many pro-inflammatory markers were also found to contribute to renal damage, such as interleukin-6 (IL-6) and tumor necrosis factor-alpha (TNF-α). TNF-α is a key pro-inflammatory cytokine which contributes to glomerular inflammation and renal fibrosis [6]. Additionally, inflammatory mediators such as TNF-α decrease peroxisome proliferator-activated receptor-γ coactivator-1α (PGC-1α) expression which is a transcriptional coactivator and main regulator for mitochondrial biogenesis that synchronizes the transcriptional process to increase mitochondrial mass hence permitting adaptation of tissues to conditions with increased energetic needs [7]. Mitochondrial biogenesis is a multifaceted process involving synthesis of mitochondrial membranes and encoded proteins as well as replication of mitochondrial DNA (mtDNA) [8]. PGC-1α dysregulation has been connected to several inflammatory disorders and its vital role in the regulation of oxidative stress, mitochondrial function as well as metabolic pathways in miscellaneous tissues is being investigated [9–11].

Apoptosis and autophagy are stimulated by severe stress and they both participate in maintaining cell homeostasis. The anticancer effect of MTX is via apoptosis induction in cancer cells [12]. Regrettably, it may cause apoptosis induction in healthy kidney tissues. Mitochondrial membrane permeability is securely controlled by the Bcl-2 family of proteins. This family includes pro-apoptotic members such as Bax and anti-apoptotic members such as Bcl-2. They control cell death by regulating the cytochrome c release from the mitochondria [13]. On the other hand, autophagy is an essentially conserved process that eliminates needless, unhealthy or dysfunctional constituents throughout a lysosome-dependent regulated mechanism. Beclin-1 and microtubule-associated protein 1 light chain 3 (LC-3) are two main autophagy-related proteins [14]. In contradiction of LC-3, a marker of final autophagosome formation, Beclin-1 contributes to the early phases of autophagy [15]. While basal levels of induced autophagy are protective by increasing cell survival, excessive activation due to chemical stress for example may be involved in cellular injury and toxicity [16]. Few studies have indicated that ROS may play a critical role in MTX‐induced apoptosis and autophagy [12].

INF, a monoclonal antibody, is an anti-TNF-α used widely in several inflammatory diseases treatment by blocking TNF-α [17]. In this context, INF may be of value providing protection against MTX-induced nephrotoxicity. Therefore, the present study was designed to investigate the role of INF against MTX-induced nephrotoxicity and for studying the fundamental mechanisms by exploring its effect on oxidative stress, inflammation, mitochondrial biogenesis as well as Bax/Bcl2 and beclin-1/LC-3 pathways.

## Materials and Methods

### Animals

Adult male Sprague-Dawley rats (150–200 g) were purchased from the animal house facility, National Research Center, Giza, Egypt. Rats were kept at 22 to 24 °C with alternating 12 h light-dark cycles and given standardized food pellets and water ad libitum and left one week for adaptation. The study was carried out according to the Guide for Care and Use of Laboratory Animals (NIH, 1985). The study was also approved by the Research Ethics Committee, Faculty of Pharmacy, Ain Shams University, Cairo, Egypt.

### Drugs and Chemicals

MTX injection (50 mg/2 ml) from Mylan N.V, in addition to INF (Remicade®; Janssen Biotech Inc) were purchased. Highest purity grade of all chemicals and solvents were used.

### Experimental Design

Rats were randomly divided into four groups (*n* = 10/group) and treated as follows: The first group served as the control group and received saline. The second group served as MTX group and received MTX once in a dose 20 mg/kg/i.p. The third group served as INF group and received only INF in a dose of 7 mg/kg/i.p once. The fourth group served as INF + MTX group and received INF (7 mg/kg/i.p once) 3 days before a single MTX (20 mg/kg/i.p). The used doses of MTX and INF were based on the previous studies [18–21].

Five days after methotrexate injection, blood samples from the retro-orbital plexus were obtained after light anesthesia for assessing the levels of serum urea and creatinine. Finally, rats were euthanized by cervical dislocation [22, 23] and kidney tissues were dissected. Kidney samples were taken and fixed in 10% formalin for paraffin blocks preparation which were then used for histological examination and immunohistochemical analyses. The remaining kidney tissues were homogenized in saline for assessing biochemical markers. Biochemical analyses were done in duplicates and the average was taken.

### Histopathological Examination

Paraffin tissue blocks were sliced using sledge microtome at 3 microns thickness and stained by hematoxylin and eosin stain for histopathological examination by light microscope. Histopathological changes were reported by a score ranging from 0 to 4 as following: 0 (no lesions), 1 (mild), 2 (moderate), 3 (severe) and 4 (very severe lesions).

### Assessment of Serum Urea and Creatinine Levels

Serum urea and creatinine levels were measured by kits from Bio-diagnostics company, Giza, Egypt, following their standard procedures.

### Assessment of Renal Malondialdehyde (MDA) and Superoxide Dismutase (SOD)

Quantitative measurement of MDA and SOD level in renal homogenate of different groups was carried out using kits purchased from Bio-diagnostics, Giza, Egypt, following their standard procedures.

### Assessment of Renal TNF–α & IL-6

Quantitative measurement of TNF-α (Cat# E0764Ra) and IL-6 (Cat# E0135Ra) concentration in renal homogenate of different groups was carried out using ELISA assay kits purchased from Bioassay Technology, China, following the manufacturer’s instructions.

### Assessment of Renal PGC-1α Level

ELISA assay kit purchased from Bioassay Technology, China, was used for quantitative measurement of PGC-1α (Cat# E2088Ra) in renal homogenate of different groups.

### Quantitative Immunohistochemical Analysis of Bax, Bcl-2, Beclin-1 and LC-3

Using one of the following primary antibodies: mouse Bax monoclonal antibody (Elabscience, Cat# E-AB-22212), mouse Bcl2 monoclonal antibody (Elabscience, Cat# E-AB-22004), rabbit Beclin-1 polyclonal antibody (Elabscience, Cat# E-AB-53242) and rabbit LC-3 Polyclonal antibody (Thermo-Fisher Scientific, Cat# PA1-16931), immunohistochemical staining was performed according to the manufacturer’s protocol. Immunohistochemical quantification was performed using image analysis software (ImageJ, 1.48a, NIH, USA).

### Statistical Analysis

Results are expressed as mean ± SEM. One-way ANOVA followed by Tukey-Kramer test was performed. All statistical analyses were carried out using IBM© SPSS© Statistics version 23 (IBM© Corp., Armonk, NY). *P* < 0.05 is considered statistically significant.

## Results

### Infliximab ameliorated MTX-induced renal histopathological changes

**Fig. 1:**
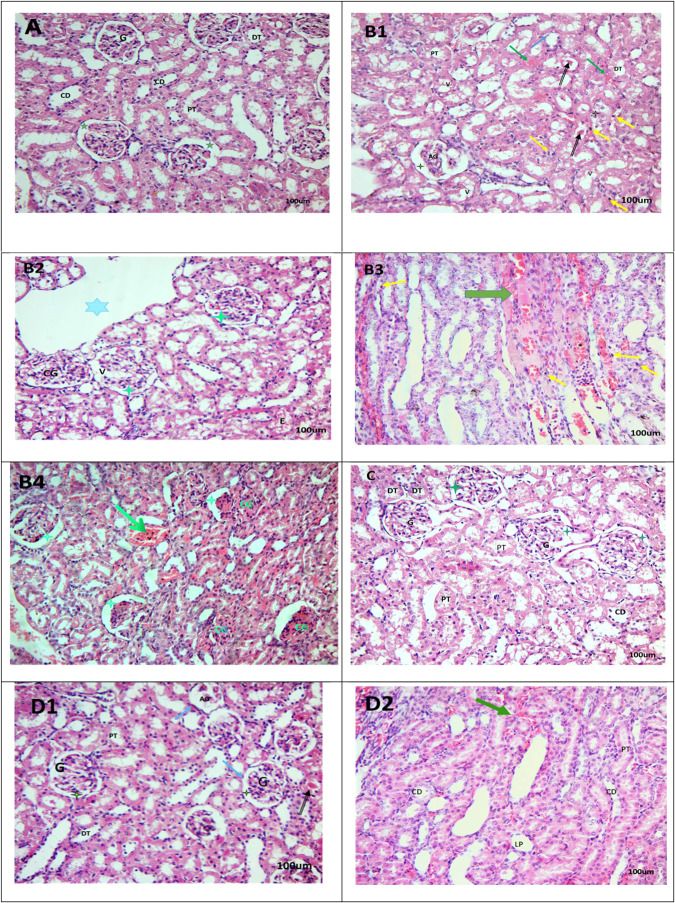
Effect of INF treatment on MTX-induced histopathological deterioration in kidneys of rats. Photomicrograph of kidney tissue section; control group (**A**): showing normal renal glomeruli (G) surrounded by visceral and parietal layers of Bowman’s capsule and separated by Bowman’s space (green star). Note proximal convoluted tubule (PT), distal convoluted tubules (DT) and collecting duct (CD) (H&E X 100, 200). MTX group (**B1**–**B4**): showing cystic luminal dilatation in some tubules (blue star), multiple pyknotic darkly stained nuclei (yellow arrow), atrophic renal glomeruli (AG) with widening of Bowman’s space (green star), some renal convoluted tubules containing homogenous acidophilic material (black arrow) in their lumens, convoluted tubules displaying cytoplasmic vacuolation (V), interstitial edema (E) and large area of hemorrhage and congested blood vessels (green arrow) were demonstrated (H&E X 200, 400). INF group (**C**): showing normal renal tubules and glomeruli (H&E X 200). INF + MTX group (**D1**, **D2**): showing apparently normal glomeruli (G) with normal Bowman’s space (green star). Note proximal convoluted tubule (PT), distal convoluted tubules (DT), collecting duct (CD) and loop of Henle (LP). Some renal convoluted tubules containing homogenous acidophilic material (black arrow) in their lumens, atrophic renal glomeruli (AG) and tiny areas of hemorrhage were still present (H&E X 100, 200)

Figure 1A (control group) shows normal structure of renal corpuscles, capillary tufts and Bowman’s capsule. The distal convoluted tubules are lined by small cuboidal cells with eosinophilic cytoplasm and have wider lumen than the proximal convoluted tubules which are lined by cuboidal cells with more eosinophilic cytoplasm and apical microvilli. The MTX group kidney tissue section revealed massive damage of renal cortex, some glomeruli showed hypercellularity, congestion of capillary tufts and vacuolated cells accompanied by narrowing of Bowman’s space, while some glomeruli appeared atrophic with widening of Bowman’s space accompanied by cyst formation. Moreover, swelling of tubular epithelial lining, tubular lumen occlusion by homogenous acidophilic material and cellular and interstitial edema as well as congested interstitial blood vessels and large areas of hemorrhage and finally tubular epithelial cell necrosis and apoptosis could be noticed (Fig. 1B1, B2, B3, B4). The INF treated group (Fig. 1C) shows normal renal tubules and glomeruli structure. Finally, INF + MTX group reveals variable degree of improvement as compared to the MTX treated group. Some glomeruli appear normally surrounded by visceral and parietal layers of Bowman’s capsule with normal Bowman’s space, but a few atrophic renal glomeruli also appeared with congestion of some glomerular tufts and tubular epithelial cell necrosis and apoptosis and smaller areas of hemorrhage (Fig. 1D1, D2). The histopathological scores (scale 0–4) of renal lesions in the kidneys of studied groups are represented in Table 1.

**Table 1:** Histopathological scores (scale 0–4) of renal lesions in the kidneys of studied groups

Histological changes	Control group	MTX group	INF group	INF + MTX group
Damaged glomeruli	0.14 ± 0.14	3.43 ± 0.3^a^	0.57 ± 0.3^b^	2.00 ± 0.22^a,b,c^
Luminal casts	0.14 ± 0.14	3.71 ± 0.18^a^	0.71 ± 0.29^b^	1.71 ± 0.18^a,b,c^
Hemorrhage	0.29 ± 0.18	3.43 ± 0.3^a^	0.43 ± 0.2^b^	2.14 ± 0.14^a,b,c^
Disorganized tubules	0.14 ± 0.14	3.29 ± 0.36^a^	0.71 ± 0.29^b^	1.86 ± 0.26^a,b^
Pyknotic darkly stained nuclei	0.14 ± 0.14	3.71 ± 0.18^a^	0.86 ± 0.34^b^	1.71 ± 0.18^a,b^
Cellular vaculation	0.29 ± 0.18	3.57 ± 0.3^a^	1.14 ± 0.34^b^	1.86 ± 0.26^a,b^

Results expressed as mean ± SEM (*n* = 10)

^a^Statistically significant from control group at *P* < 0.001

^b^Statistically significant from MTX group at *P* < 0.001

^c^Statistically significant from INF group at *P* < 0.05

### Infliximab Protected Against MTX-induced Nephrotoxicity

**Fig. 2:**
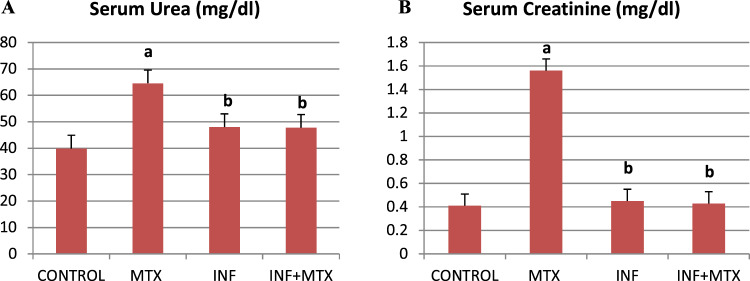
Effect of INF on serum urea and creatinine in MTX-induced nephrotoxicity in rats. **A** Serum urea. **B** Serum creatinine. Results expressed as mean ± SEM (*n* = 10). a: Statistically significant from control group at *P* < 0.001. b: Statistically significant from MTX group at *P* < 0.001

MTX injection induced 61.87 and 280.48% increase in serum levels of urea and creatinine respectively, when compared to the control group. Conversely, INF pre-treatment significantly reduced both urea and creatinine serum levels by 26.05 and 72.43% respectively, as compared to the MTX group. It should be noted that INF pre-treatment almost returned kidney function tests to the basal levels. Rats injected with INF only didn’t show significant difference as compared to the control group (Fig. 2).

### Infliximab Abrogated MTX-induced Renal Oxidative Stress

Renal MDA level was significantly elevated in the MTX group by 46.93% as compared with the control group. In contrast, the INF pre-treatment group showed significantly less MDA level by 20.69% when compared to the MTX group as represented in Table 2. Meanwhile, a single dose injection of MTX significantly reduced renal antioxidant SOD level by 29.57% as compared to the control group, whereas INF co-treatment induced significant escalation in SOD level by 23.01% as compared to the MTX group (Table 2).

**Table 2:** Effect of INF on renal MDA & SOD level in MTX-induced nephrotoxicity in rats

Treated groups	Renal MDA (nmole/mg protein)	Renal SOD (U/mg protein)
CONTROL	3.75 ± 0.08	14.81 ± 0.12
MTX	5.51 ± 0.37^a^	10.43 ± 0.73^a^
INF	4.36 ± 0.14^b^	13.74 ± 0.49^b^
INF + MTX	4.37 ± 0.16^b^	12.83 ± 0.67^b^

Results expressed as mean ± SEM (*n* = 10)

^a^Statistically significant from control group at *P* < 0.001

^b^Statistically significant from MTX group at *P* < 0.001

### Infliximab Ameliorated MTX-induced Renal Inflammatory Markers

**Fig. 3:**
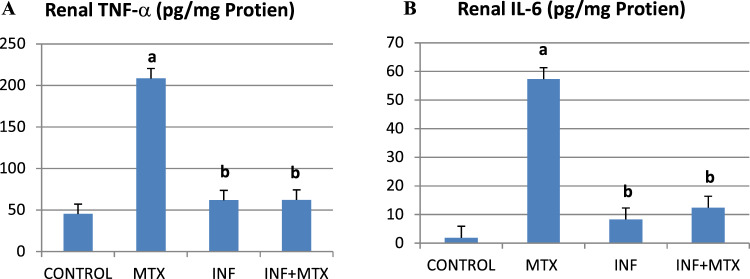
Effect of INF on renal TNF-α and IL-6 in MTX-induced nephrotoxicity in rats. **A** TNF-α, **B** IL-6. Results expressed as mean ± SEM (*n* = 10). a: Statistically significant from control group at *P* < 0.001. b: Statistically significant from MTX group at *P* < 0.001

Renal tissue levels of TNF-α and IL-6 were noticeably elevated in MTX-injected group by 361.06 and 2915.79% respectively, as compared to the control group (Fig. 3). Nevertheless, INF pre-treatment before MTX injection considerably ameliorated this effect. As compared to the MTX group, INF pre-treatment significantly reduced the levels of TNF-α and IL-6 by 70.11 and 78.36% respectively.

### Infliximab Augments Renal PGC-1α level in MTX-induced Nephrotoxicity

The normal control value for renal PGC-1α was 4.51 ± 0.31 pg/mg protein. MTX injection significantly reduced PGC-1α renal levels by 73.83%, as compared to the control group. Interestingly, pre-treatment with INF showed a significant elevation in PGC-1α level by 198.31%, as compared to the group that received MTX only (Table 3).

**Table 3:** Effect of INF on renal PGC-1α level in MTX-induced nephrotoxicity in rats

Treated groups	Renal PGC-1α (pg/mg protein)
CONTROL	4.51 ± 0.31
MTX	1.18 ± 0.06^a^
INF	4.02 ± 0.33^b^
INF + MTX	3.52 ± 0.29^b^

Results expressed as mean ± SEM (*n* = 10)

^a^Statistically significant from control group at *P* < 0.001

^b^Statistically significant from MTX group at *P* < 0.001

### Infliximab Attenuated MTX-induced Renal Apoptosis

**Fig. 4:**
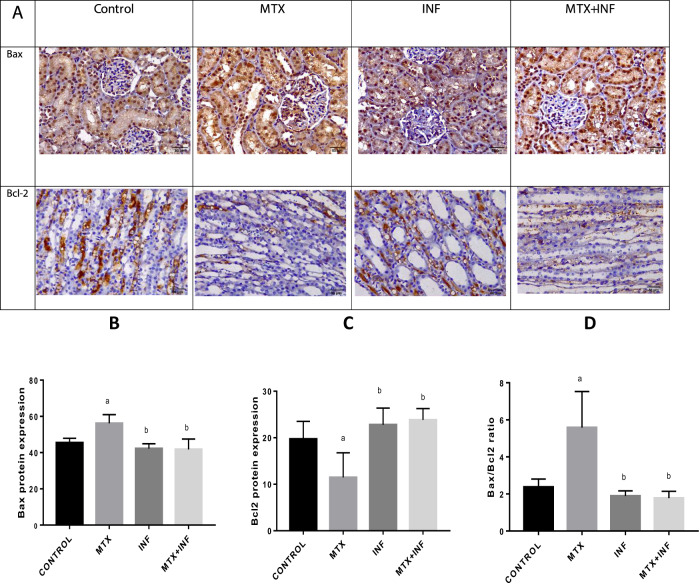
Effect of INF treatment on renal Bax and Bcl2 protein expression in MTX-induced nephrotoxicity in rats. **A** Immunohistochemical staining of renal Bax and Bcl-2 expression. **B**, **C** Quantitative image analysis for Bax and Bcl-2 immunohistochemical staining. **D** Quantitative image analysis for Bax/Bcl-2 ratio. Results expressed as mean ± SEM (*n* = 10). a: Statistically significant from control group at *P* < 0.001; b: Statistically significant from MTX group at *P* < 0.001

Assessment of MTX-induced apoptotic changes was carried out through Bax and Bcl-2 proteins’ immunohistochemical examination (Fig. 4A). MTX significantly increased pro-apoptotic Bax expression by 23.59% along with decreased expression of anti-apoptotic Bcl-2 by 41.89% as compared to control group. On the contrary, INF pre-treatment ameliorated MTX intoxication by significantly downregulating Bax expression and upregulating the expression of Bcl-2 (Fig. 4B, C). Moreover, semi-quantitative analysis of performed immunostaining demonstrated significantly elevated Bax/Bcl-2 ratio in the MTX group compared to control group. Whereas significantly reduced Bax/Bcl-2 ratio as compared to the MTX group was detected in INF pre-treatment group (Fig. 4D).

### Infliximab Attenuated MTX-induced Renal Autophagy

**Fig. 5:**
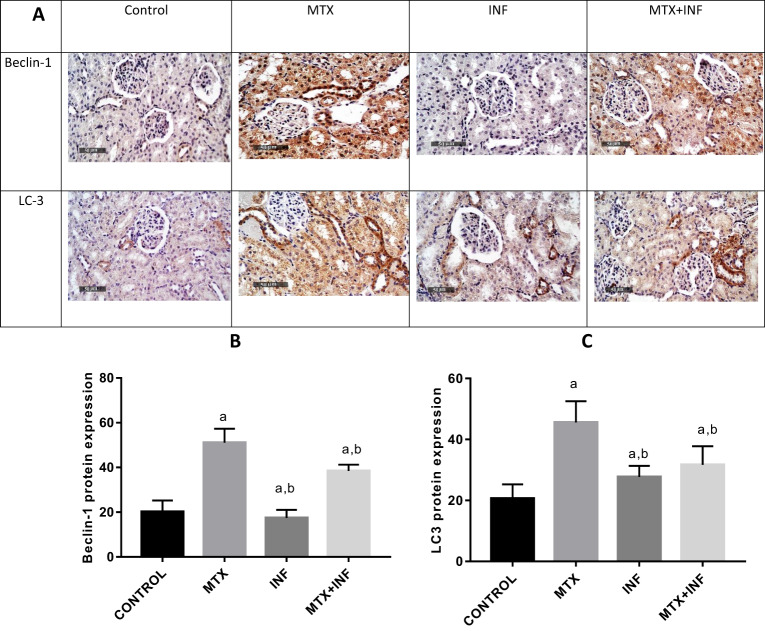
Effect of INF treatment on renal Beclin-1 and LC-3 protein expression in MTX-induced nephrotoxicity in rats. **A** Immunohistochemical staining of renal Beclin-1 and LC-3 expression. **B**, **C** Quantitative image analysis for Beclin-1 and LC-3 immunohistochemical staining. Results expressed as mean ± SEM (*n* = 10). a: Statistically significant from control group at *P* < 0.001. b: Statistically significant from MTX group at *P* < 0.001

MTX administration switched on autophagic response in renal tissue manifested by a significant increase in the protein expression of beclin-1 and LC-3 by 153.57 and 120.84%, respectively as compared to the control group. On the other hand, INF pre-treatment significantly reduced beclin-1 and LC-3 protein expression by 24.8 and 30.49%, respectively as compared to the MTX group and thus alleviated the heightened autophagic response (Fig. 5A–C).

## Discussion

Presently there is urgent need to overcome the side effects of MTX therapy. Multiple organ toxicities take place after using high MTX chemotherapy doses. Even though the exact mechanism is not well revealed, MTX-induced nephrotoxicity is still a main source of concern, thus restraining its wide use in clinical practice. MTX-induced nephrotoxicity may be direct, caused by its precipitation in the renal tubules or may be indirect by inducing oxidative stress, inflammation, and apoptosis [4]. We hereby studied the antioxidant and anti-inflammatory potentials of the anti-TNF-α, INF, as well as its prospective impact on mitochondrial biogenesis, apoptosis and autophagy in MTX-induced nephrotoxicity model.

MTX-injected rats showed a marked increase in kidney function indices: serum urea and creatinine, as compared to control rats. Acute nephrotoxicity was further proved through histopathological examination by monitoring renal casts, congestion, large areas of hemorrhage as well as cell necrosis and apoptosis. Many antineoplastic drugs and their metabolites are eliminated through kidneys, making tubules vulnerable to damage. Thus, this functional impairment in kidneys may be due to precipitation of MTX in renal tubules [24]. Notably, this renal damage was greatly improved by INF pre-treatment. This was demonstrated by significant amelioration in the nephrotoxicity indices and MTX-induced renal histopathological changes, thus indicating the nephroprotective property exerted by INF against the nephrotoxic impacts of MTX. Kirbas et al. previously displayed that INF may provide nephroprotection against MTX in rats by affecting carbonic anhydrase-II enzyme activities and inhibiting purine metabolism [19]; however, they did not cover all the underlying mechanisms deeply.

The complete mechanism underlying MTX nephrotoxicity is not well clarified. MTX may trigger production of reactive oxygen species (ROS) or reactive nitrogen species (RNS), thus inducing oxidative stress [25]. Interestingly, PGC-1α is the master modulator of mitochondrial biogenesis and function, including ROS detoxification and regulation of mitochondrial antioxidant enzymes expression such as SOD [26]. The present study confirmed the essential role of PGC-1α dysregulation and oxidative stress in the mechanism of MTX-induced nephrotoxicity as proved by considerable decline of renal tissue PGC-1α and SOD antioxidant as well as marked elevation in renal tissue level of the lipid peroxidation index; MDA. Our findings further showed that INF administration significantly induced renal PGC-1α level and attenuated MTX-induced renal oxidative stress. Interestingly, to the best we know, this is the first study reporting that INF anti-oxidative effect is mediated through PGC-1α expression induction.

The pro-inflammatory response triggered by MTX was evident by assessment of renal inflammatory markers. MTX injection turned on a vigorous inflammatory response in renal tissue which was evident by a considerable boost in both IL-6 and TNF-α, thus suggesting the involvement of proinflammation in MTX-induced renal damage. It is worth mentioning that activation of oxidative stress is a main contributor to this MTX-induced inflammation by activating NF-κB, a redox responsive transcription factor, which consequently provokes transcription of several inflammatory cytokines [27, 28]. Conversely, INF attenuated the inflammatory response induced by MTX, which may be explained by its ability to counteract TNF-α pathway thus decreasing the expression of inflammatory cytokines [29]. These results demonstrate the antinflammatory beneficial effect of INF on the kidney in this model together with halting the MTX-induced oxidative stress. In agreement with our results, the study of Zălar et al. demonstrated that INF increases the antioxidant capacity and reduces both oxidative stress and inflammation by inhibiting the TNF-α cascade, causing decreased IL-1β and IL-6 cytokines [30].

Oxidative stress and inflammation work in concert inducing MTX‐associated apoptosis and autophagy. It is noteworthy that at subcellular levels, mitochondria are regarded as primary targets of MTX toxicity [12, 31]. MTX-induced ROS considerably disrupt mitochondrial structure and function, thus initiating translocation of p53 and promoting cytochrome c leakage and cell death via apoptosis [13, 32, 33]. The study of Xiong et al. revealed that ROS mediated MTX-induced apoptosis and autophagy in a spermatocyte cell line [12]. Additionally, the study of Aslankoc et al. demonstrated MTX-mediated induction of apoptosis as well as increased beclin-1 and ATG12 mRNA expressions in the cerebral cortex tissue of MTX-treated rats [34]. These studies are in agreement with our results where MTX triggered both renal apoptosis and autophagy, showing significantly reduced renal anti-apoptotic bcl-2, increased renal pro-apoptotic Bax expression and Bax/bcl-2 ratio, along with significantly up-regulated expression of both autophagy-related proteins; beclin-1 and LC-3. Therefore, induction of apoptosis and autophagy could be considered among the main contributing factors of MTX‐induced nephrotoxicity. On the contrary, INF pre-treatment inhibited the signaling cascade of apoptosis as confirmed by induced anti-apoptotic Bcl-2 protein expression and inhibited pro-apoptotic Bax protein expression as well as restoration of Bax/Bcl-2 ratio to normal level in the kidney of MTX-injected rats. Besides, INF was also capable of ameliorating MTX-induced autophagy as clarified by down-regulating both beclin-1 and LC-3 expression. Due to the accredited role of oxidative stress and inflammation in inducing apoptosis and autophagy [12, 31], the anti-apoptotic and anti-autophagic effect of INF could be directly related to its antioxidant and anti-inflammatory capacity. Interestingly, this is the first study reporting the anti-autophagic effect of INF in MTX-induced nephrotoxicity model.

## Conclusion

The present study revealed the promising nephroprotective action of INF against MTX-induced nephrotoxicity, principally throughout its anti-oxidative, anti-inflammatory, anti-autophagic and anti-apoptotic effects, plus its capability to augment mitochondrial biogenesis. Furthermore, our study suggests PGC-1α as a prospective therapeutic target in reducing MTX-induced nephrotoxicity.

## Data Availability

All data generated or analyzed during this study are included in this published article [and its supplementary information files].
